# (-)-OSU6162 reduces freezing elicited by context-conditioned fear in rats

**DOI:** 10.1177/02698811251389543

**Published:** 2025-11-29

**Authors:** Daniela Atanasovski, Sven Melker Hagsäter, Marcus Myrehag, Karl Arheden, Sandra Sjöblad, Elias Eriksson

**Affiliations:** 1 Department of Pharmacology, Institute of Neuroscience and Physiology, Sahlgrenska Academy, University of Gothenburg, Sweden

**Keywords:** OSU6162, amphetamine, dopamine D2 receptor, sigma receptor, 5-HT2A receptor

## Abstract

**Objective:**

(-)-OSU6162 is an antagonist at dopaminergic D_2_ receptors which – like other D_2_ antagonists – dampens spontaneous activity in animals exploring a novel environment. However, unlike other D_2_ antagonists, (-)-OSU6162 has unexpectedly been found to stimulate locomotor activity in inactive, habituated rodents. To what extent the compound may increase activity also in other situations characterised by reduced locomotion is unknown, and it has also not been clarified whether two other receptors also targeted by (-)-OSU6162 – serotonergic 5-HT_2A_ receptors and sigma-1 receptors – are involved in its unusual behavioural profile. The objective of the present study was to investigate the possible impact of (-)-OSU6162 on the pronounced inactivity in the form of freezing displayed by rats exposed to context-conditioned fear. For comparison, the effect of amphetamine – another dopamine-augmenting and activating compound – was also explored in the same paradigm.

**Methods:**

The impact of (-)-OSU6162 and amphetamine on the expression of freezing was assessed in rats exposed to contextual fear conditioning using electrical foot shocks. It was also assessed whether the increase in activity observed in animals treated with (-)-OSU6162 could be countered by pretreatment with a D_2_/D_3_ antagonist (raclopride), a 5-HT_2A_ inverse agonist (MDL100907), or a sigma-1 receptor antagonist (BD1063).

**Results:**

While (-)-OSU6162 markedly reduced freezing behaviour, amphetamine abolished it completely. The effect of (-)-OSU6162 was countered by raclopride but neither by MDL100907 nor by BD1063.

**Conclusion:**

(-)-OSU6162 reduces the expression of context-conditioned fear displayed as freezing by a mechanism involving D_2_ but not 5-HT_2A_ or sigma-1 receptors.

## Introduction

The so-called dopamine stabiliser (-)-OSU6162 has been shown to modulate rodent activity in an atypical manner (Rung et al., 2008). In line with being an antagonist at dopaminergic D_2_ receptors (Natesan et al., 2006), it causes inhibition of locomotor activity in active animals exploring a novel environment or treated with amphetamine. Unlike other D_2_ antagonists, however, (-)-OSU6162 does not elicit catalepsy even at high doses in rodents (Natesan et al., 2006) and does not cause extrapyramidal side effects in humans (Nilsson et al., 2020; Tolboom et al., 2015). Also, unlike other D_2_ antagonists, (-)-OSU6162 was unexpectedly shown to cause locomotor stimulation in animals habituated to a testing arena (Carlsson et al., 2011; Natesan et al., 2006). Striatal D_2_ receptor occupancy displayed by (-)-OSU6162 appears relatively high in rodents (Natesan et al., 2006) and non-human primates (Ekesbo et al., 1999) but more modest in humans (Tolboom et al., 2015).

In addition to being a D_2_ receptor ligand, (-)-OSU6162 also displays affinity to serotonergic 5-HT_2A_ and 5-HT_2B_ receptors (acting as partial agonist) (Carlsson et al., 2011), high affinity to the sigma-1 receptor (Sahlholm et al., 2013), and relatively low affinity to dopaminergic D_3_ receptors and serotonergic 5HT_1A_ and 5HT_1D_ receptors, respectively (Burstein et al., 2011). In contrast, it has little or no affinity to adrenergic, histaminergic, or other dopaminergic receptors, and no affinity to the noradrenaline, dopamine, or serotonin transporters (Burstein et al., 2011).

Given the involvement of dopamine in numerous psychiatric and neurological conditions, the possible impact on spontaneous activity of a compound displaying this highly unusual pharmacological profile deserves further attention, especially since (-)-OSU6162 is entirely devoid of the side effects marring D2 receptor antagonists used for psychosis, hyperprolactinemia being the sole exception (Nilsson et al., 2020). Likewise, it lacks the side effects of central stimulants and appears not to display abuse liabilities (Asth et al., 2021).

Rats re-introduced to a neutral context in which they have previously been exposed to an aversive stimulus in the form of foot shocks display context-conditioned fear expressed as freezing, *that is*, a marked reduction of spontaneous locomotor activity (Grillon, 2002). The aim of the present study being to explore if a stimulatory impact of (-)-OSU6162 on locomotion may also be revealed in another situation than that of animals being inactive after having habituated to their cage, we studied the possible impact of this molecule on freezing resulting from context-conditioned fear and compared it to that of a dopamine releaser, amphetamine. After having observed that (-)-OSU6162 does stimulate locomotion in this paradigm – as does amphetamine – we assessed to what extent the effect of the former may be countered by antagonists at D_2_/D_3_ (raclopride), 5-HT_2A_ (MDL100907), and sigma-1 (BD1063) receptors, respectively. To exclude the possibility of reduced freezing behaviour following (-)-OSU6162 being merely a reflection of the well-known stimulatory impact of the drug in habituated rodents, we also assessed the extent to which (-)-OSU6162 might activate rats exposed to the conditioning chamber one week prior to the acute experiment without receiving foot shocks.

## Material and methods

### Animals

A total of 243 male Sprague Dawley rats (Janvier, Le Genest-Saint-Isle, France) were used in this study. Animals were 8–9 weeks upon arrival and housed in groups of 3 per cage. They were maintained on a 12-hour light/dark cycle, had access to food and water *ad libitum*, and were housed in 21 ± 1^o^C with 50% humidity. No animal participated in more than one experiment. All experiments followed the legislative directive 2010/63/EU of the European Union and were approved under permit nr 2354/20 and 4122/21 by the regional animal ethics committee at the University of Gothenburg in accordance with the guidelines of the Swedish Board of Agriculture.

### Drugs

(-)-OSU6162 was gifted from Arvid Carlsson Research AB (Gothenburg, Sweden), S(-)-raclopride (+)-tartrate salt, and D-amphetamine sulfate were purchased from Merck KGaA (Darmstadt, Germany), and MDL100907 and BD1063 dihydrochloride were purchased from Tocris (Bristol, UK). All drugs were dissolved in 0.9% saline on the day of the experiment and given subcutaneously in volumes of 2 ml/kg with the exception of MDL100907, which was dissolved in a few drops of hydrochloric acid, made up to volume with 0.9% saline, and buffered to pH > 5.5 with sodium hydroxide. BD1063 was given at a dose of 15 mg/kg which was selected based on pilot experiments following a survey of the relevant literature; for example, BD1063 at a dose range of 3–30 mg/kg has been shown to reduce alcohol-seeking behaviour (Blasio et al., 2015; Sabino et al., 2009), reduce binge-like eating (Cottone et al., 2012), and to counteract MDMA-induced (but not spontaneous) motor activity (Brammer et al., 2006), while lower doses of 7.5–10 mg/kg have shown to specifically counteract sigma-1 receptor induced increase of substance intake (Hiranita et al., 2010; Sabino et al., 2011). MDL100907 was given at a dose of 1 mg/kg, which is a dose that in our laboratory has been shown to effectively antagonise 5-HT_2A_ receptor agonist-induced reduction of contextual fear conditioning (Hagsater et al., 2021) and which, according to numerous previous studies, should be sufficient to block other 5-HT_2A_-mediated effects (Carbonaro et al., 2015). The dose of raclopride was chosen following a pilot experiment investigating dose-response effects on (-)-OSU6162-induced and spontaneous locomotion (see section “Discussion”).

### Contextual fear conditioning

The contextual fear conditioning experiments were performed using the contextual near-infrared Video Fear Conditioning System for Rat (Med Associates, Sant Albans, VT, USA). The conditioning chamber was enclosed by a sound-attenuating cubicle in which a constant 60-dB white background noise was delivered during all experimental sessions. In the fear acqusition session, rats were allowed to habituate for five minutes prior to receiving five electric foot shocks (0.6 mA, 30 seconds inter-shock interval). Seven days later, the animals were administered (-)-OSU6162, amphetamine, or saline 40 minutes before they were re-exposed to the chamber for five minutes during which contextual fear-conditioned freezing was assessed. In other experiments, antagonists at D_2_, 5-HT_2A_, or sigma-1 receptors, or saline were administered 10 minutes prior to (-)-OSU6162 or saline. Freezing was calculated by automatic scoring of video recordings (immoblity > 1 second) and presented as the % time the animal spent immobile during the five-minute test (Hagsater et al., 2019). Normally, animals subjected to a novel cage display considerable exploratory activity – in the present study, gross observation (though without quantification) was applied to confirm that immobile animals displayed typical signs of freezing, that is, rigged tail, arched back, erected fur, and retracted ears, rather than any unusual form of inactivity not pertaining to the typical freezing characteristics. In a control experiment aimed at addressing the possible impact of (-)-OSU6162 and raclopride, respectively, on spontaneous activity in animals not displaying freezing, rats were exposed twice to the same context as above but without receiving foot shocks. Faeces trays were always emptied, and the wire mesh cage was cleaned using 70% ethanol before every new test session.

### Locomotor activity

In order to assess if the notably low dose of raclopride (0.03 mg/kg) found to counter the effect of (-)-OSU6162 in the freezing paradigm could also prevent the locomotor stimulation elicited by (-)-OSU6162 in habituated animals, rats were placed in the centre of ventilated and sound-attenuated motron boxes illuminated by a dim light (40 × 40 cm, Med Associates Inc., Fairfax, VT, USA) and allowed to habituate for 30 minutes before being administered raclopride followed 10 minutes later by (-)-OSU6162 after which the motor activity of the animals was assessed for 30 minutes. Movement was registered as ambulatory counts caused by crossing a square pattern of infrared beams scattered across the floor.

### Statistical analysis

Data were analysed, and graphs were created using GraphPad Prism version 10.2.3 (Boston, MA, USA) and Microsoft Excel version 16.97 (Redmond, WA, USA). Assessment of normality was undertaken using the Shapiro-Wilk test. Comparisons between groups were performed with one-way analysis of variance (ANOVA) when normally distributed, or the Kruskal-Wallis test when not, followed by the Benjamini-Hochberg procedure. The significance threshold was set to 0.05. For all ANOVAs, homogeneity of variance was estimated using Levene’s test. Normally distributed data are displayed as means + standard errors of the mean (SEM), while data not normally distributed are displayed as medians + interquartile ranges (IQR).

## Results

**Figure 1.**
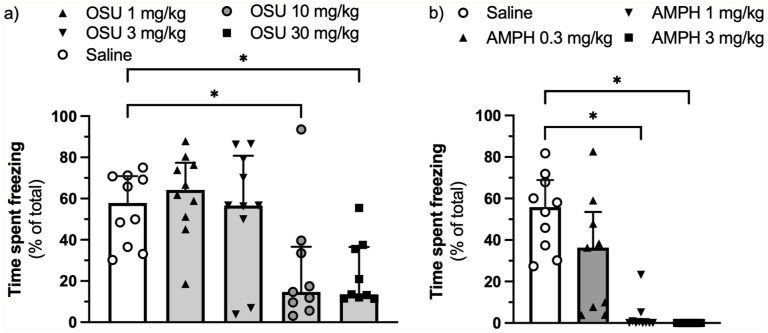
Effect of (-)-OSU6162 (a) and AMPH (b) on the expression of context-conditioned fear assessed as percentage of time spent freezing. AMPH: Amphetamine; IQR: Interquartile ranges. *n* = 9–10 per group. Values are displayed as median + IQR. *p < 0.05.

(-)-OSU6162 at doses of 10 and 30 mg/kg – but not at doses of 1 or 3 mg/kg – reduced the expression of contextual fear-conditioned freezing (H(4) = 16.23, η^²^ = 0.284, *p* = 0.003, Figure 1(a)). Amphetamine at doses of 1 and 3 mg/kg abolished the expression of freezing (H(3) = 30.54, η^²^ = 0.787, *p* < 0.0001, Figure 1(b)).

**Figure 2.**
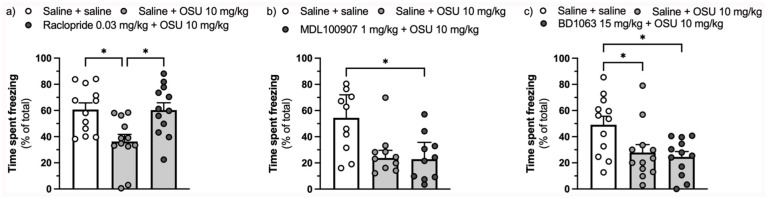
Effect of pretreatment with the D_2_/D_3_ receptor antagonist raclopride (0.03 mg/kg) (a), the 5-HT_2A_ receptor inverse agonist MDL100907 (1 mg/kg) (b), and the sigma-1 receptor antagonist BD1063 (15 mg/kg) (c) on the reduction in the expression of context-conditioned fear assessed as percentage of time spent freezing caused by (-)-OSU6162 (10 mg/kg). SEM: Standard errors of the mean; IQR: Interquartile ranges. *n* = 10–12 per group. Values are displayed as mean + SEM (a and c) or median + IQR (b). *p < 0.05.

The D_2_/D_3_ antagonist raclopride (0.03 mg/kg) (F(2,33) = 6.793, ηp^²^ = 0.292, *p* = 0.003, Figure 2(a)), but neither the 5-HT_2A_ inverse agonist MDL100907 (1 mg/kg) (H(2) = 7.388, η^²^ = 0.200, *p* = 0.025, Figure 2(b)) nor the sigma-1 antagonist BD1063 (15 mg/kg) (F(2,33) = 5.546, ηp^²^ = 0.252, *p* = 0.008, Figure 2(c)), prevented the impact of (-)-OSU6162 on freezing.

**Figure 3.**
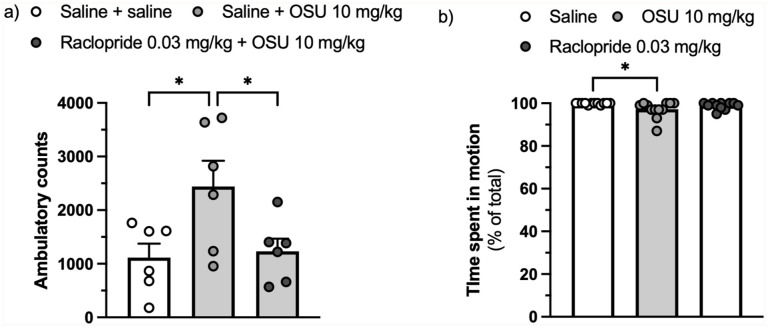
Effect of the D_2_/D_3_ receptor antagonist raclopride (0.03 mg/kg) on the increase in locomotion (ambulatory counts) caused by (-)-OSU6162 (10 mg/kg) in rats habituated to a locomotor activity arena. *n* = 6 per group. Values are displayed as mean + SEM (a) Effect of (-)-OSU6162 (10 mg/kg) or raclopride (0.03 mg/kg) on the time spent in motion displayed by animals re-exposed to a conditioning chamber that they had been exposed to 1 week earlier without receiving foot shocks. *n* = 12 per group. Values are displayed as median + IQR (b). SEM: Standard errors of the mean; IQR: Interquartile ranges. *p < 0.05.

The same (low) dose of raclopride (0.03 mg/kg) that was found to antagonise the (-)-OSU6162-induced reduction in freezing also countered the stimulatory effect of (-)-OSU6162 in rats habituated to a locomotor activity arena (F(2,15) = 4.583, ηp^²^ = 0.379, *p* = 0.028, Figure 3(a)) but did not reduce spontaneous locomotion in rats subjected a second time to a conditioning chamber to which they had been exposed a week before but without receiving foot shocks (H(2) = 8.447, η^²^ = 0.195, *p* = 0.015, Figure 3(b)). In the same experiment, (-)-OSU6162 exerted a very modest but significant reduction in locomotion.

## Discussion

While amphetamine usually stimulates locomotion in rodents, regardless of their baseline activity, the impact of (-)-OSU6162 is more complex. Thus, while the drug dampens locomotion in active, exploring animals – as would be expected from a D_2_ receptor antagonist – it has been found to stimulate locomotion in habituated, non-active animals (Carlsson et al., 2011). While an activating impact of (-)-OSU6162 has previously been demonstrated in this situation only, we now report that a robust stimulatory effect of (-)-OSU6162 is at hand also in rats displaying a lack of motion when being exposed to context-conditioned fear. The activating effect of (-)-OSU6162 was blocked by a low dose of the selective D_2_/D_3_ antagonist raclopride but neither by a 5-HT_2A_ antagonist nor by a sigma-1 receptor antagonist.

The activating effect of (-)-OSU6162 in rats displaying freezing at the second exposure to the conditioning chamber was not merely a reflection of the previously reported increase in activity in rats habituated to their chamber. Thus, animals exposed to the same conditioning chamber twice, but without having experienced foot shocks at their first visit, displayed no signs of habituation when re-introduced to the chamber, and their activity was dampened rather than stimulated by (-)-OSU6162.

While it cannot be excluded that the freezing-reducing effect of (-)-OSU6162 is caused by impaired memory retrieval, this seems unlikely since the drug has been attributed no negative impact on memory, but – on the contrary – been shown to prolong object location memory as well as to reverse memory impairments following scopolamine administration in mice (Nilsson and Carlsson, 2013). Likewise, (-)-OSU6162 has been reported not to impair cognitive function in humans (Khemiri et al., 2020).

The dose of the selective D_2_/D_3_ receptor antagonist raclopride presently shown to prevent the activating effect of (-)-OSU6162 has previously been reported too low to impact motor activity *per se* (Adams et al., 2001; Hillegaart and Ahlenius, 1987). Likewise, this dose of raclopride did not impact spontaneous locomotion in animals exposed a second time to the conditioning chamber without having received foot shocks at their first visit. The effect of raclopride on (-)-OSU6162-induced activation of fear-conditioned animals hence does not appear to be merely the result of physiological antagonism. On the other hand, the same dose was found to counter not only the stimulatory effect of (-)-OSU6162 in rats displaying freezing but also the locomotor stimulation produced by (-)-OSU6162 when administered to animals habituated to the testing arena.

The anti-freezing effect of (-)-OSU6162 being effectively countered by a low dose of raclopride suggests an involvement of D_2_/D_3_ receptors. A possible explanation to why the stimulatory effect of a D_2_/D_3_ antagonist, (-)-OSU6162, is antagonised by another D_2_/D_3_ antagonist, raclopride, might be that (-)-OSU6162 increases extracellular levels of dopamine by preferentially blocking autoreceptors, hence causing an indirect stimulation of postsynaptic locomotion-stimulating D_2_/D_3_ receptors not blocked by (-)-OSU6162 but by a low dose of raclopride. In line with this possibility, in vivo microdialysis experiments have revealed (-)-OSU6162 to increase extracellular levels of dopamine in the rat striatum (Steensland et al., 2012). Needless to say, this mechanism could be relevant also for the stimulatory effect of (-)-OSU6162 in habituated rodents.

It was to address this possibility we compared the effect of (-)-OSU6162 to that of an indirect D_2_ receptor agonist, amphetamine, using the same paradigm and the same setting. In line with the possibility of (-)-OSU6162 reducing freezing by increasing extracellular levels of dopamine, the central stimulant also reduced freezing but to an even greater extent. Thus, while the maximum reduction in the expression of freezing after (-)-OSU6162 was approximately 85%, high doses of amphetamine eliminated the freezing reaction entirely, and even rendered the animals hyperactive, as judged by gross observation. Such a discrepancy is not counterintuitive since (-)-OSU6162 and amphetamine are likely to elicit an increase in extracellular dopamine levels by different mechanisms: autoreceptor blockade and reversal of the direction of the dopamine transporter, respectively. Likewise, it is compatible with the notion of (-)-OSU6162 acting as a dopamine *stabiliser*, while amphetamine, in contrast, is a forceful dopamine augmenter. While we have not identified any previous study showing the impact of systemic amphetamine on context-conditioned fear manifested as freezing, local administration of the drug in the prefrontal cortex of rats has been reported to reduce freezing in an auditory fear-conditioning paradigm (Babaei et al., 2011). Systemic administration of D_2_ agonists have been reported to reduce freezing caused by context-conditioned fear, but follow-up experiments using local application of the drug in the ventral tegmental area, where dopaminergic cell bodies are situated, led to the suggestion that this effect be caused by an autoreceptor-mediated reduction in dopamine release rather than a postsynaptic effect (de Souza Caetano et al., 2013). In the same study, the D_2_ antagonist sulpiride administered systemically or locally into the basolateral amygdala also reduced freezing.

While the observed antagonism between (-)-OSU6162 and raclopride may thus tentatively be explained by (-)-OSU6162 enhancing extracellular levels of dopamine by blocking D_2_ autoreceptors, a different possibility should also be considered. There are hence some indications that (-)-OSU6162 – instead of being merely a silent antagonist at the orthostatic site of the D_2_ receptor – may also act as a positive allosteric modulator at D_2_ receptors (and/or to exert limited intrinsic activity upon the same); until now there is, however, only *in vitro* evidence supporting this possibility (Kara et al., 2010; Lahti et al., 2007). Hence, while allosteric activation could possibly account for the activating effects of the compound, for example, in rats displaying freezing behaviour, orthosteric antagonism may be responsible for its inhibiting effects. Moreover, (-)-OSU6162 could tentatively be driving the formation of heteroreceptor complexes comprising D_2_ and, for example, sigma-1 receptors, as previously demonstrated in brain tissue (Borroto-Escuela et al., 2020), or complexes comprising D_2_ and 5-HT_2A_ receptors (Borroto-Escuela et al., 2010). However, as discussed in the next paragraph, neither a 5-HT_2A_ receptor antagonist nor a sigma-1 receptor antagonist could block the effect of (-)-OSU6162.

Apart from its impact on D_2_ receptors, (-)-OSU6162 is also a partial agonist at 5-HT_2A_ receptors. We have previously, using the same experimental setting, found two 5-HT_2A_ receptor agonists – psilocybin and 25CN-NBOH – to reduce the expression of conditioned fear, and shown these effects to be effectively blocked by the 5-HT_2A_ antagonist/inverse agonist MDL100907 (Hagsater et al., 2021). Also, it has been shown that MDL100907 blocks the (-)-OSU6162-induced stimulation of locomotion elicited in mice rendered monoamine-depleted by means of pretreatment with reserpine, a response that could not be blocked by D_2_ antagonism (Carlsson et al., 2011). It was therefore deemed important to explore the possible impact of MDL100907 at a dose effectively blocking 5-HT_2A_ receptors (Carlsson et al., 2011; Hagsater et al., 2021) on the freezing-reducing effect of (-)-OSU6162. The response being the same in animals pretreated with MDL100907 as in those pretreated with saline, however, argues against 5-HT_2A_ receptor activation being involved in the studied effect of (-)-OSU6162.

Previous reports suggest that sigma-1 receptor agonists reduce the expression of freezing (Noda et al., 2000). (-)-OSU6162 displaying high affinity for this receptor (Sahlholm et al., 2013), thus prompted an attempt to block the effect of (-)-OSU6162 on freezing also with a sigma-1 receptor antagonist, BD1063, at a dose previously shown to effectively block the sigma-1 binding site (Brammer et al., 2006). (-)-OSU6162, however, reduced freezing to the same extent also after pretreatment with this sigma-1 antagonist.

While the primary aim of the present study was to shed further light on the enigmatic stimulatory impact of (-)-OSU6162 on locomotor activity in rat, it should be noted that contextual fear conditioning has often been used as a putative animal model of human anxiety (Grillon, 2002; Hagsater et al., 2019; Izumi et al., 1999). The possibility that (-)-OSU6162 reduced freezing in the present experiments by reducing fear, rather than by a non-specific stimulation of locomotor activity, should thus not be ruled out (de Souza Caetano et al., 2013). The compound has not displayed effects similar to those of drugs for anxiety in the elevated plus maze (Studer et al., 2016) but did exert such an effect in anxiety-prone Flinder rats exposed to the novelty suppressed feeding test (Melas et al., 2021).

In conclusion, the results of this study add to the growing body of data suggesting that the D_2_ receptor antagonist (-)-OSU6162 impacts dopamine transmission in an unusual manner. The drug displaying both similarities and differences when compared to other dopamine-modulating substances, such as D_2_ receptor antagonists, D_2_ receptor agonists, and dopamine releasers, warrants further exploration of its possible use for various dopamine-related clinical conditions.
